# Epilepsy and amygdalar enlargement: cause or consequence?

**DOI:** 10.1055/s-0046-1825522

**Published:** 2026-07-14

**Authors:** Cristian Hardaman, Luis Ariel Miquelini, Federico Sánchez González, Juliana Belvel Fernandes Lastoria, Yemina Soledad Neme Segura, Ignacio Lagger, Oscar Martínez

**Affiliations:** 1 Hospital de Agudos Dr Ramón Madariaga, Neurology Service, Misiones, Argentina.; 2 Hospital Británico, Department of Neurology, Buenos Aires, Argentina.; 3 Hospital Italiano, Department of Diagnostic Imaging, Buenos Aires, Argentina.; 4 Hospital de Clínicas José de San Martín, Department of Neurology, Buenos Aires, Argentina.; 5 Pontificia Universidad Católica Argentina, Consejo Nacional de Investigaciones Científicas y Técnicas, Instituto de Investigaciones Biomédicas, Buenos Aires, Argentina.

**Keywords:** Epilepsy, Temporal Lobe, Amygdala, Hypertrophy, Seizures

## Abstract

The amygdala is a central structure of the limbic system. For several decades, volumetric asymmetries of the amygdala have been described, initially associated with psychiatric disorders. In epilepsy, a growing literature highlights the distinctive features of amygdala enlargement (AE), considering that since its original description, diagnosis has been primarily radiological. The objective of the present work is to demonstrate, through reviews of existing academic publications and through our experience, that its function goes beyond imaging, contemplating a defined clinical, electrographic, and histopathological pattern that responds to both medical and surgical treatment. Therefore, it would be an entity that extends beyond a reactive phenomenon. In conclusion, AE would be a specific syndrome defined with particular characteristics that extends beyond a reactive mechanism secondary to adjacent epileptogenic networks.

## INTRODUCTION

The amygdala, first described by Burdach in the early 19 ^th^ century, is located deep within the temporal lobe. 1 It forms part of the limbic system and accounts for approximately 0.3% of the total brain volume. 2 For many years, the amygdala was considered primarily the brain's “fear center.” More recent work has shown that it integrates experiences with their consequences, programs appropriate behavioral responses, shapes affective behavior, and modulates mood in response to external stimuli. 3 In particular, it is critically involved in emotional learning and processing, especially in the expression of fear and anger. 4

## METHODS

A descriptive literature review was conducted based on systematic search and critical analysis of scientific reports focusing on amygdalar enlargement (AE) as a diagnostic subgroup within temporal lobe epilepsy (TLE). Clinical features, neurophysiology, neuroimaging findings, and responses to medical and surgical treatment were examined.

Sources were identified through searches of electronic health science databases, including PubMed, MEDLINE, and SciELO. Keywords such as *temporal lobe epilepsy* , *amygdala enlargement* , *neuroimaging* , and *structural findings* were used. Case reports, cohort studies, literature reviews, and original articles specifically addressing AE in patients with TLE were included. Epilepsies unrelated to the temporal lobe, editorials lacking scientific evidence, and articles with unclear methodology were excluded.

Initially, titles and abstracts were screened. Subsequently, articles were classified according to descriptive findings, and full texts underwent critical review. A descriptive comparative analysis was then performed to identify common patterns across studies, allowing a more refined interpretation of AE as a diagnostic entity.

## ANATOMICAL RELATIONS OF THE AMYGDALA

The temporal lobe has four surfaces: the lateral, medial, superior (or opercular), and inferior (or basal) surfaces.

Its medial surface presents complex microanatomy and maintains close relationships with intraventricular structures, including the amygdala and hippocampus 5 .

The depth of the Sylvian fissure, known as the Sylvian cistern, contains two compartments: the sphenoidal and the operculoinsular. The sphenoidal compartment lies between the carotid cistern medially, the posterior orbitofrontal area superiorly, the polar plane of the temporal lobe laterally, and the amygdala inferiorly.

**Figure 1:**
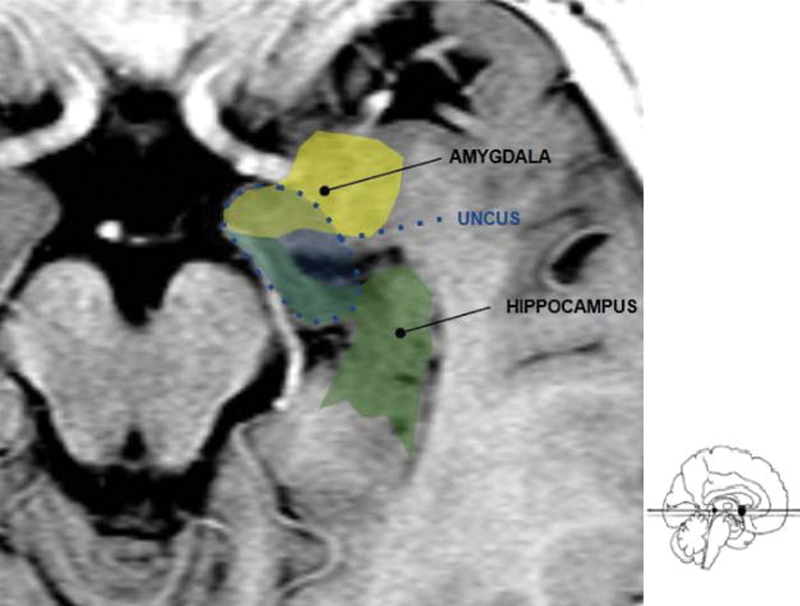
Axial section showing the anatomical relationship of the amygdala (highlighted in yellow) with intraventricular structures, and the hippocampus (in green). The uncus, containing the semilunar and ambient gyri, is shown in dotted outline. (Author's property).

The amygdala forms the anterior wall of the temporal horn of the lateral ventricle and occupies most of the anterior segment of the uncus 6 ( Figure 1 ). The anterior uncus contains the semilunar and the ambient gyri; the semilunar gyrus overlies the cortical nucleus of the amygdala. The inferior fronto-occipital fasciculus (IFOF) is a major white matter tract that serves as a critical connection between the frontal lobe and posterior brain regions, including the parietal, occipital, and temporal lobes. Its temporal component, referred to as the deep or ventral portion, is located inferior to the uncinate fasciculus (UF), together forming the temporal trunk. 7 The UF, forming the anterior portion of the temporal stem, interconnects the orbitofrontal cortex with the anterior temporal lobe. It extends through the extreme and external capsules, entering the limen insulae to connect the orbitofrontal cortex with the temporal pole and amygdala.

## THE AMYGDALOID COMPLEX

Morphologically, the amygdala comprises 13 distinct nuclei, each with specific connections, functions, and relationships, collectively termed the amygdaloid complex.

Based on cytoarchitectonic organization, histochemistry, and connectivity, this complex is divided into three main groups: a deep or basolateral group, a superficial or cortical group, and a centromedial group. 8 Murphy et al. (1987) demonstrated that the human amygdaloid complex is normally symmetrical. 9 In 1992, Watson et al. attempted to establish anatomical guidelines for delineating the hippocampus and amygdala using low-field magnetic resonance imaging (MRI), though boundaries were difficult to define. 10 With the advent of high resolution MRI, the anatomical limits of the amygdala have become more clearly delineated. Automated segmentation of 7.0 Tesla MRI, aided by next-generation multi-atlas frameworks and auto-context models, has enabled increasingly precise volumetric characterization. 11

## AMYGDALAR ENLARGEMENT

With the advent of neuroimaging, asymmetries in amygdalar volume began to be recognized. Initially, AE was associated with psychiatric comorbidities. Early studies reported abnormalities in the size of the amygdala and hippocampus in young women with major depressive disorder receiving antidepressant treatment. In this context, enlargement of the left amygdala has been shown to predict impaired emotional memory and heightened anxiety symptoms. 12 Increasingly consistent evidence indicates that individuals with social anxiety exhibit increased bilateral amygdalar volume and enlargement of the left hippocampus. This finding has been attributed to the role of limbic structures in processing contextual signals and classifying stimuli as potentially threatening, leading to early amygdalar hyper-responsiveness. Such changes may reflect increased metabolic activity and regional blood flow, potentially resulting in subtle volumetric enlargement. 13

Morphological abnormalities have also been described in the amygdala of individuals with psychopathy, likely related to reorganization of the nuclei within the amygdaloid complex. 14 One of the earliest studies documenting a significant increase in amygdalar volume in patients with epilepsy related psychosis was reported by Tebartz van Elst, establishing a link between amygdalar pathology, psychiatric disorders, and epilepsy. 15 This observation highlighted a clear overlap between psychobiological factors and epileptogenesis.

The tendency to identify AE is greater in non-lesional TLE compared with TLE with mesial temporal sclerosis (MTS). In the latter, the amygdalae typically appear normal in volume or reduced due to the sclerotic process. 16

## EPILEPTOGENIC NETWORKS OF THE AMYGDALA

The amygdala plays a central role within epileptogenic networks. Owing to its dense connectivity with cortical and subcortical regions, it is positioned as a key structure for both the generation and propagation of ictal activity. Functionally, it is integrated into circuits responsible for emotional modulation, memory, and vigilance, 17 reinforcing its relevance in the pathophysiology of mesial TLE. 18 19

**Figure 2:**
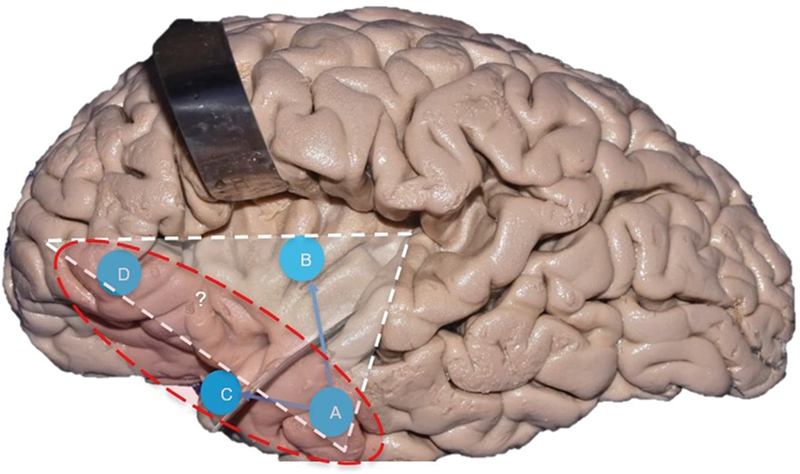
Common amygdala-centered epileptogenic networks: (A–B and A–C) frequently observed; (A–B–D, triangular dashed outline) and (D–A–C, ovoid dashed outline) less common without clear predominance. (A: temporal lobe; B: insula; C: mesial region; D: frontal lobe). (Author's property).

According to the epileptogenicity index, 20 four principal organizational patterns of amygdala-centred epileptogenic networks have been identified: the most frequent is the temporo-insular network, followed by the temporo-mesial network. Less commonly, temporo-insular-prefrontal and prefrontal-temporo-mesial networks are observed, with no clear predominance between the latter two. 21 ( Figure 2 )

Dysfunction within these networks contributes not only to the emergence and spread of seizures, but also to neurocognitive deficits during the interictal period. Connections with the anterior insula, for example, facilitate propagation of epileptic activity to regions implicated in visceral and autonomic manifestations, which are characteristic of mesial seizure semiology. By contrast, connections with the hippocampus and other mesial limbic structures reinforce excitatory circuits that contribute to chronicity and spread of epileptiform activity. 22

Projections to the prefrontal cortex are particularly relevant for attention, consciousness, and executive control. Alterations in these pathways are associated with impaired executive function, diminished inhibitory control, and emotional dysregulation. 23

Furthermore, the involvement of thalamic nuclei and the ascending reticular activating system suggest that, under conditions of hyperexcitability or enlargement, the amygdala may contribute directly to the disturbances of consciousness observed during seizures. 24

## AMYGDALAR ENLARGEMENT AS A SUBGROUP

For many years, it has been shown that amygdala volume is significantly increased on the side of seizure onset in patients with epilepsy, often in parallel with hippocampal volume changes in the same region. More recent studies have described a heterogeneous condition characterized by MRI findings of an enlarged amygdala with a normal hippocampal volume—termed hippocampal-negative AE. 16

It remains unclear whether AE is a marker of local epileptogenicity or a nonspecific finding; however, accumulating evidence supports its recognition as a potential subgroup. Recent studies have reported AE in a substantial proportion of patients with MRI-negative TLE, with prevalence rates ranging from 12 to 64%. 25

This has led to the proposal of a distinct clinical entity: epilepsy with AE syndrome. Within this framework, TLE may not represent a single pathological entity but rather a spectrum of heterogeneous subtypes, each involving independent yet interconnected epileptogenic structures that manifest with overlapping but distinct clinical characteristics. 26

At present, it is also uncertain whether AE is more commonly associated with hippocampal-negative or non-lesional TLE, or whether it should instead be considered part of an extra-temporal epileptic syndrome. 27

## DEFINITION OF AE

The major limitation in defining AE is the lack of consensus regarding volumetric thresholds or measurement parameters. Reported volumetric ranges for AE are broad, extending from 1,700 mm ^3^ to 3,194 mm ^3^ , compared with mean normal volumes of 1,240 mm ^3^ to 2,146 mm ^3^ . 9 10 11 12 13 14 15 16 17 18 19 20 21 22 23 24 25 26 27 28 29 Operational definitions vary across studies. Some define AE as a normalized amygdala volume more than two standard deviations above the mean, corrected for total brain volume. Others use an interhemispheric asymmetry > 6% relative to controls as a diagnostic threshold. Asymmetries below this threshold are typically classified as asymmetrical AE without significant clinical implications. 30

## CLASSIFICATION BY AMYGDALAR MORPHOMETRY

In many cases, discrepancies between volumetric findings and visual assessment reflect morphological variations in the shape of the amygdala, which depend on the arrangement of its nuclei. Based on morphometric characteristics, two principal patterns have been described. When enlargement is oriented perpendicular to the brain's longitudinal axis, it is classified as an eccentric amygdala. In contrast, when enlargement predominates along the brain's longitudinal axis, it is referred to as an elongated amygdala. 31

To date, no significant correlation has been demonstrated between amygdalar morphometry and the organization of epileptogenic networks, nor between amygdalar volume and the specific cortical regions involved. Nevertheless, increasingly complex epileptogenic networks have been described, in some cases extending well beyond the temporal lobe. 32

## ICTAL SEMIOLOGICAL FEATURES

Since the earliest reports, ictal semiology associated with AE has included ictal fear, gastrointestinal sensations, and autonomic manifestations, described respectively by Cendes, Weiser, and Biraben. 33 Over time, it has become more difficult to identify a pure semiological pattern, as more recent studies describe a broad spectrum of clinical manifestations that only partially overlap with the original descriptions. 34

The most frequently reported symptoms include lateralized painful paresthesias, sensations of heat or burning, ascending epigastric aura, dizziness or floating sensations, and intense anxiety or fear. Auditory illusions, euphoria, a sense of wellbeing, experiential déjà vu, and unpleasant anticipatory feelings have also been reported, together with autonomic manifestations such as facial pallor, palpitations, and gastric discomfort.

Emotional and behavioral changes, including dysthymia, nervousness, irritability, and aggressive behavior, may also occur. In some patients, motor auras manifest with oral or manual automatisms. 35

Despite this variability, a consistent hallmark across reports in impaired consciousness during the ictal event, which is thought to reflect the engagement of adjacent epileptogenic networks. Another feature that appears to characterize this condition is the later age of seizure onset compared with other forms of epilepsy. This has been hypothesized to result from developmental anomalies or from local neuroinflammatory processes, although the precise mechanisms underlying this delayed clinical presentation remain incompletely understood. 36 37

## IMAGING DIAGNOSIS

Magnetic resonance imaging is the imaging modality of choice for diagnosing AE. The typical radiological pattern includes asymmetry of the amygdalae with subtle enlargement on the affected side, accompanied by mild T2 and fluid-attenuated inversion recovery (FLAIR) hyperintensity, slightly reduced T1 signal, and variable findings on diffusion-weighted imaging and apparent diffusion coefficient (ADC) maps, depending on the proximity to the most recent seizure. Careful review of so called “negative” MRIs is warranted, with particular attention to these subtle changes.

**Figure 3:**
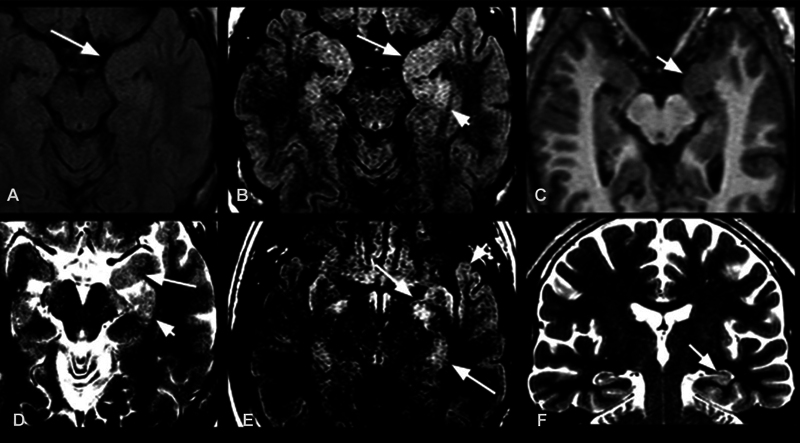
Brain MRI showing mild enlargement of the left amygdala with subtle FLAIR and T2 hyperintensity (A: standard window). Findings are more evident when window settings are adjusted (arrows in B and D). T1 sequence shows minimal hypointensity (C). The left hippocampus demonstrates FLAIR and T2 hyperintensity (short arrow in D; long arrows in E and F). (Author's property).

Adjusting the window width and level settings can enhance detection of such abnormalities ( Figure 3 ).

Additional signal and volume changes may also occur in the hippocampus, including FLAIR and T2 hyperintensity with volume loss, as well as in the cortex and white matter of the temporal lobe, which may present with more subtle FLAIR and T2 hyperintensity. These findings are consistent with cases of AE associated with MTS. 38 39

## HISTOPATHOLOGICAL FINDINGS

Histopathological analyses of amygdalae resected during epilepsy surgery have revealed a range of abnormalities. The most frequently reported is focal cortical dysplasia, followed by gliosis and low-grade tumours, most often gangliogliomas, but also including oligodendrogliomas and astrocytomas. Importantly, these pathological findings were largely indistinguishable from imaging studies performed prior to surgery, underscoring the limitations of structural imaging in predicting microscopic pathology. 9 25 26 27 28 29 30 31 32 33 34 35 36 37 38 39

## ELECTROENCEPHALOGRAPHY

Few studies have specifically addressed the electroencephalographic features of AE. Video-electroencephalogram (EEG) monitoring has shown that ictal onset may arise from the enlarged amygdala as well as the hippocampus in all reported cases. Sterelectroencephalography (SEEG) has further confirmed that the ictal onset zone frequently involves not only the hippocampus but also the enlarged amygdala, a finding consistent with classic MTS, in which periodic spike-and-wave discharges are typically observed on the affected side. 40

**Figure 4:**
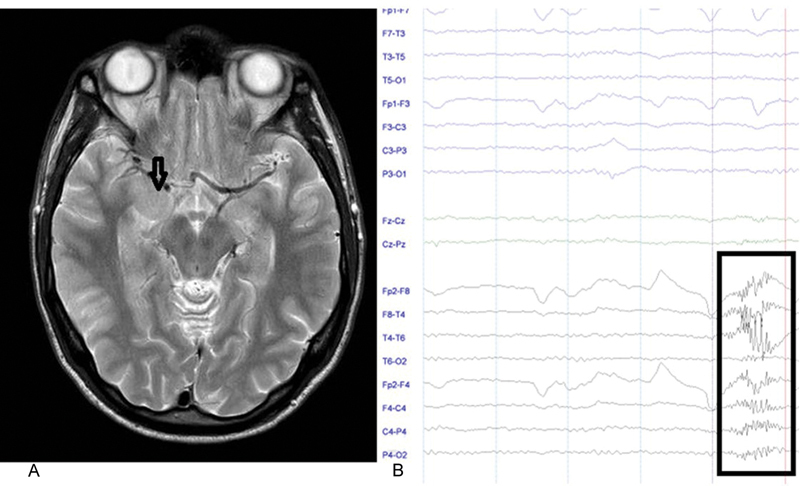
A: Axial T2-weighted brain MRI demonstrating right amygdalar enlargement (black arrow). B: Scalp EEG of the same patient showing bursts of short-interval polyspikes with a focus in regions F8 to T4 and T4 to T6 (inset). (Author's property).

In contrast, bursts of polyspike discharges have been described as the characteristic EEG pattern in patients with TLE and AE without MTS 41 ( Figure 4 ). Although the precise pathophysiological mechanism underlying this pattern remains unclear, the observation supports the concept that AE may display an electrophysiological behavior distinct from MTS. Interictal epileptiform discharges in TLE with AE generally correlate with the ictal onset zone, reinforcing the possibility that the enlarged amygdala itself may serve as the primary epileptogenic focus, though seizure foci in adjacent regions cannot be excluded. 42

## RESPONSE TO PHARMACOLOGICAL TREATMENT

Follow-up studies of patients with TLE and AE who responded favorably to antiseizure medication (ASM) 43 from the onset of seizures and remained seizure free have shown progressive reduction in amygdala volume over 1 to 2 years. This contrasted with patients experiencing frequent seizures or poor drug response, in whom amygdalar volume remained unchanged. 28

In general, patients with AE demonstrate good responsiveness to ASM. The underlying mechanism for volumetric reduction is not fully understood, but neuronal hypotrophy secondary to epileptogenic processes has been proposed. The temporal evolution of these imaging changes suggests that MRI could serve as a potential predictive marker of treatment response and as a tool for monitoring long-term outcomes. 29

Although additional studies are needed to confirm these findings, the correlation between seizure control and amygdala volume reduction has been consistently demonstrated in multiple controlled series. 28 43 44

## RESPONSE TO SURGICAL TREATMENT

Although the functional role of the amygdala in TLE is well established, the specific significance of AE remains incompletely defined. Evidence from surgical series indicates that patients with pharmacoresistant epilepsy who undergo extended temporal lobectomy including both the hippocampus and amygdala achieve better seizure outcomes compared with those undergoing selective hippocampectomy, underscoring the active contribution of the amygdala to epileptogenesis. 28

**Figure 5:**
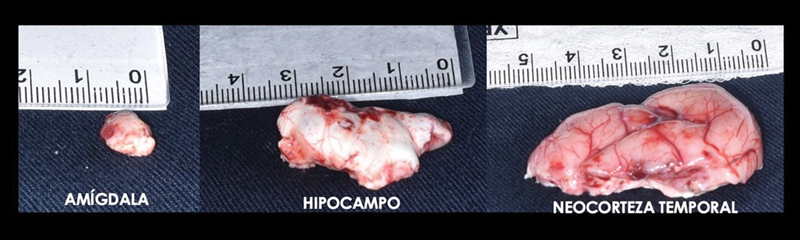
Surgical resection of temporal lobe structures including neocortex and mesial regions (amygdala and hippocampus). (Author's property).

However, the optimal surgical strategy for patients with AE and hippocampal-negative TLE remains uncertain. It is not yet clear whether selective amygdalectomy or extended amygdalohippocampectomy provides superior outcomes. In such cases, SEEG may be particularly valuable for accurately localizing the epileptogenic focus and guiding surgical decision making. 45 ( Figure 5 )

## DISCUSSION

Temporal lobe epilepsy encompasses a variety of ictal onset zones within a single structure, each sharing specific characteristics yet necessitating subclassifications for better clinical understanding. Amygdalar enlargement represents a notable exception, frequently identified on imaging in patients with epilepsy. For some, this entity should be considered a distinct subtype of TLE—epilepsy with AE syndrome—while others interpret it as a secondary reactive process related to adjacent epileptogenic structures.

A major challenge in defining AE is the lack of standardized volumetric criteria, creating methodological barriers to consistent analysis. Techniques described range from visual inspection and manual tracing with voxel counts, to voxel-based morphometry using automated tissue segmentation and advanced volumetric software. Although AE may sometimes appear as an isolated imaging finding, its recurring clinical, radiological, and pathological features suggest that it might represent the “tip of the iceberg” of a broader limbic system abnormality. 46

Some authors propose that AE reflects a reactive process secondary to recurrent seizures, characterized pathologically by gliosis and atypical neuronal clustering within the white matter, a transient postictal phenomenon. 34 Other reports suggest that the amygdala and hippocampus may serve as independent epileptogenic generators, or alternatively as structures that exhibit rapid bidirectional propagation of epileptic activity. This has led to the hypothesis that AE could represent a transitional stage toward MTS, rather than a stable condition.

The growing number of reports of AE in TLE has strengthened its recognition as a clinical syndrome. Importantly, the term “enlargement” refers only to size increase and does not necessarily imply activity. Moreover, AE can occasionally be observed incidentally in nonepileptic patients during routine brain MRI, possibly reflecting vascular hyperperfusion rather than structural predisposition, in which case it may represent an incidentaloma rather than an active pathological entity. 40

Support for considering AE as a TLE subgroup also derives from its later age of seizure onset compared with classic MTS, and from its lower incidence of childhood febrile seizures, suggesting distinct developmental and clinical pathways. 47

Proposed etiologies include secondary hypertrophy following hypoxic injury 44 or an autoimmune basis, although robust evidence linking AE to limbic encephalitis remains lacking. 34 Chronic neuroinflammatory processes, with or without self-limited progression, could also underlie the condition. 47 A “two-hit” model has been suggested, in which an initial event promotes latent epileptogenesis, and a subsequent insult triggers overt epileptogenic mechanisms, with AE emerging as the radiological correlate. 48

In practice, AE is often detected only after meticulous re-examination of MRI scans, particularly in patients with hippocampal-negative epilepsy. Subtle abnormalities must be carefully evaluated with targeted high-resolution MRI, volumetric sequences, and follow-up imaging when suspicion persists. Combined with semiological, EEG, and metabolic data, these tools may help establish AE as a marker of disease progression and treatment response. 34

In conclusion, in recent years, increasing attention has been directed toward the role of the amygdala in TLE without MTS. The constellation of clinical, imaging, and histopathological features associated with AE has been reproduced across studies, supporting its recognition as a distinctive and recurring pattern.

From a therapeutic perspective, AE appears to be responsive to pharmacological treatment, with longitudinal imaging studies demonstrating reductions in amygdalar volume over time in patients achieving seizure control. Moreover, surgical series have consistently shown favorable outcomes after extended temporal resections that include the amygdala, further underscoring its active contribution to epileptogenesis.

Taken together, these findings suggest that AE in patients with epilepsy should not be regarded merely as a reactive phenomenon. Instead, AE should be considered a clinically meaningful entity that extends beyond imaging, with implications for diagnosis, prognosis, and treatment planning.
